# A Red Flag: A Case of Oropharyngeal Cancer Masquerading as an Ulcer of an Infective Origin

**DOI:** 10.7759/cureus.50411

**Published:** 2023-12-12

**Authors:** Ahmad Firdaus Habib Rahman, Ahmad Kamil Ahmad Fahmi, Nur Eliana Ahmad Tarmizi, Chua Hui Heng, Avatar Singh Mohan Singh

**Affiliations:** 1 Otolaryngology - Head and Neck Surgery, Taiping Hospital, Taiping, MYS; 2 Pathology, Taiping Hospital, Taiping, MYS

**Keywords:** periodic follow up, p16 gene, oral ulcer, squamous cell neoplasm, oral and oropharyngeal cancer

## Abstract

Oropharyngeal squamous cell carcinoma is a prevalent neoplastic condition. The incidence rate in Malaysia is rising, with human papillomavirus (HPV) infection being recognized as a significant contributing factor. Hence, it is paramount for physicians to effectively diagnose and identify significant indicators that may indicate a malignant etiology. In this study, we present a case of a middle-aged Malay male who presented with the primary symptom of persistent right throat discomfort for one month. The preliminary presentation, blood parameters, and initial histopathological examination (HPE) findings indicate the presence of an infection. However, despite undergoing several medical treatments, the patient's symptoms remain, albeit with only minor clinical improvement. Subsequently, the patient underwent a biopsy under general anesthesia, which subsequently yielded a report indicating the presence of oropharyngeal squamous cell carcinoma with a negative p16 status. Therefore, it is imperative for clinicians to possess knowledge of warning flags and exercise vigilance when encountering a patient who fails to respond despite thorough and precise evaluation. If there is a strong suspicion of malignancy, it is imperative to do a comprehensive clinical investigation and regular monitoring.

## Introduction

The World Health Organization (WHO), in its Globocan Report 2020, estimated that the global burden of cancer in 2020 was 19.3 million new cases, 9.9 million cancer deaths, and 50 million people living with cancer within five years of diagnosis [1]. Oropharyngeal squamous cell carcinoma is a prevalent form of neoplasia, with the tongue having the highest frequency of involvement, accounting for 41.52% of cases [2-4]. The prevalence in Malaysia is increasing, with human papillomavirus (HPV) infection being identified as a contributing factor. The 2017 WHO Classification of Head and Neck Tumours edition has divided tumors of the oral cavity and oropharynx into different chapters and sub-classified according to HPV status. The prognosis of patients is significantly influenced by factors such as tumor size and spread, nodal involvement, and the presence or absence of loco-regional metastasis. Therefore, accurate diagnosis is key, and defaulting on cancer treatment can lead to adverse clinical outcomes such as progression of the disease and reduced survival.

## Case presentation

A 54-year-old male of Malay descent with no prior medical conditions and a history of smoking presented with a chief complaint of soreness in the right throat that has persisted for one month, accompanied by odynophagia. He did not exhibit any constitutional symptoms, had no significant sexual history, and did not have a family history of malignancy.

**Figure 1:**
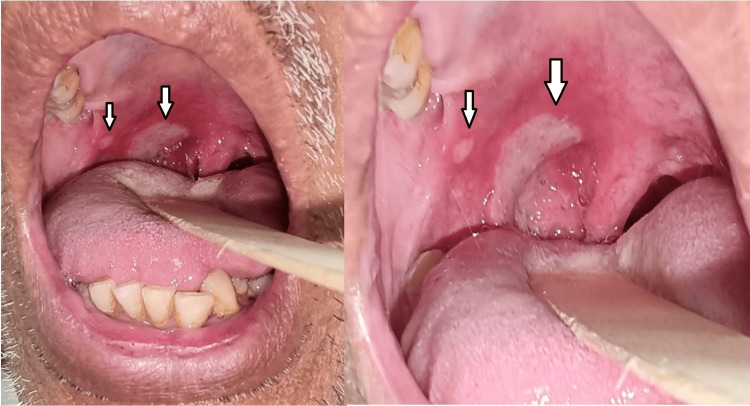
An ulcer within the right tonsil, which extended toward the right anterior pillar and posteriorly to the posterior pillar. There was also another small circular ulcer adjacent to it as shown by the arrows in the figure.

Intraoral examination revealed a 1 cm x 2 cm ulcer within the right tonsil, which extended toward the right anterior pillar and posteriorly to the posterior pillar (Figure 1). Another small ulcer was also present adjacent to the ulcer on the anterior pillar. The left tonsil is grade I and normal. Oral hygiene is unremarkable. There was no neck node palpable.

**Figure 2:**
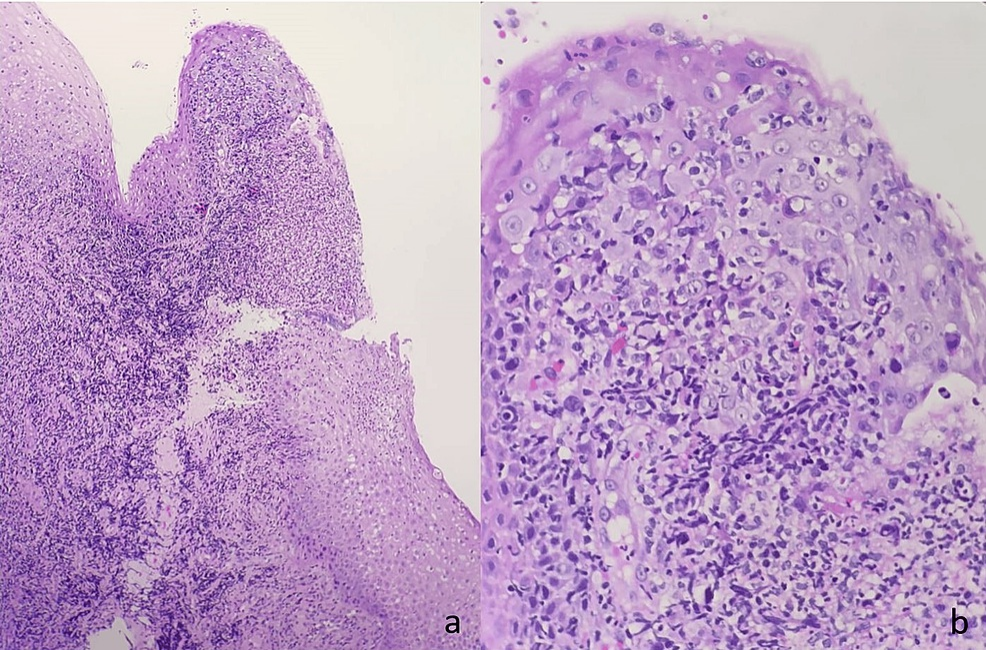
a: Acute-on-chronic inflammation. Histological findings of the tonsillar tissue covered by stratified squamous epithelium with an area of ulceration. (H&E with 100X magnification). b: Acute-on-chronic inflammation. Histological findings of the tonsillar tissue covered by stratified squamous epithelium with intraepithelial neutrophilic infiltration and focal epithelial atypia, characterized by enlarged nuclei with prominent nucleoli. (H&E with 400X magnification).

Due to the initial suspicion of malignancy, the patient underwent a biopsy (first biopsy). The biopsy specimen exhibited evidence of both acute and chronic inflammation, characterized by the presence of reactive epithelium atypia and surface ulceration (Figure 2).

The patient received multiple courses of antibiotics and antifungal medications over the follow-up period. Figure 2 shows the results of the biopsy, which indicated the presence of infectious etiologies. Furthermore, a swab for culture and sensitivity (C+S) over the ulcer site also showed Streptococcus mitis. He was treated with antibiotics in accordance with his C+S result throughout his weekly follow-up.

**Figure 3:**
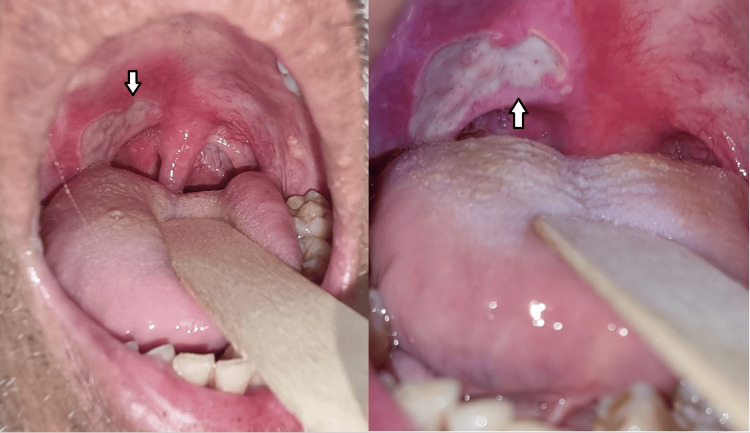
The ulcer has more defined borders and is less sloughy as shown by the arrow in the figure. Note also the small circular ulcer seen in the previous visit was absent, which indicates a sign of recovery.

He was seen in our clinic weekly to assess his oral cavity lesion progress. He received an initial week's course of Augmentin which showed no response, then a week's course of Azithromycin, which showed similar changes, followed by a week of Cefuroxime and Nystatin, which showed no response as well. He was also given oral aid and Difflam Gargle & Mouth solution (NSW, Australia). Nevertheless, despite treatment with various medications, the symptoms were persistent, and examination of the oral cavity revealed comparable clinical observations despite the therapeutic interventions (Figure 3). A repeat biopsy (second biopsy) on further follow-up showed inflamed squamous epithelium. No malignant cells were seen as well. 

**Figure 4:**
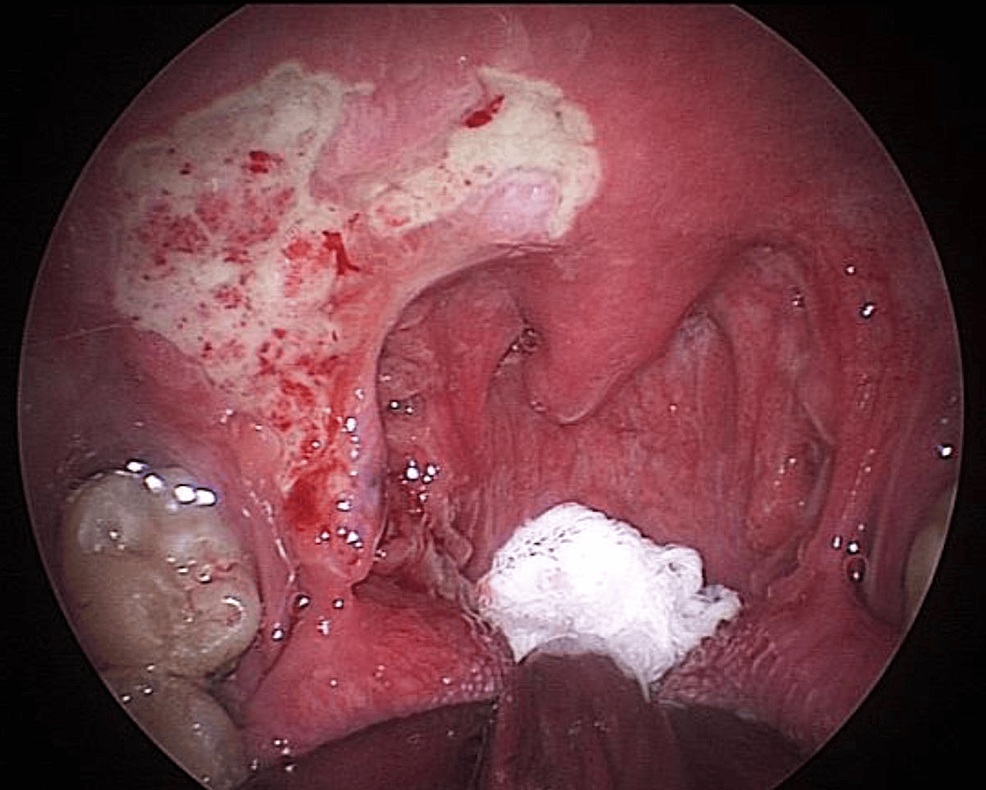
An ulcerative lesion was observed on the lower pole of the right tonsil, as well as ulcerative lesions on the soft palate to the anterior pillar.

Due to a high suspicion of malignancy and almost no response to antibiotics (even as suggested by the infective parameters in the first and second biopsies and swab C+S), he underwent examination under anesthesia, direct laryngoscopy, biopsy, and contact endoscopy (third biopsy), which was done under general anesthesia (Figure 4). During the intraoperative examination, an ulcerative lesion was observed on the lower pole of the right tonsil, as well as ulcerative lesions on the soft palate to the anterior pillar. Contact endoscopy over the right soft palate also showed type 3 vessels. Other supraglottic structures were unremarkable.

**Figure 5:**
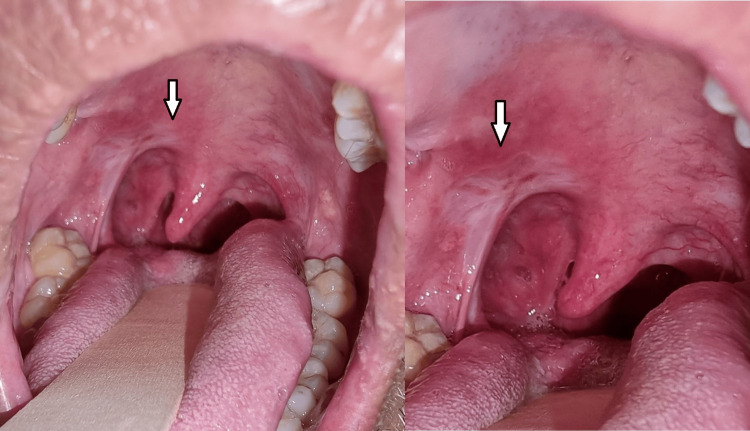
Intraoral view four weeks postoperatively Only fibrotic changes were seen over the previous biopsied sites as shown by the arrow in the figure. No more ulcers were observed.

He was monitored while waiting for his histopathological examination (HPE) results and received regular follow-up. The postoperative assessment revealed satisfactory healing, and the patient's symptoms improved. The biopsied areas healed well (Figure 5).

**Figure 6:**
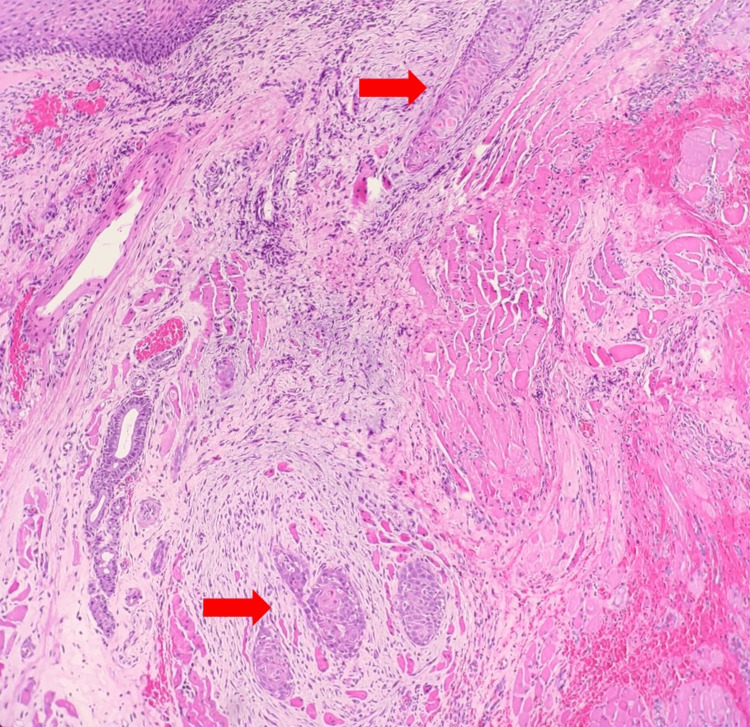
Moderately differentiated squamous cell carcinoma Histological findings of nests and trabeculae of the tumor cells invading the stroma and insinuating between the skeletal muscle bundles. (H&E with 200X magnification).

**Figure 7:**
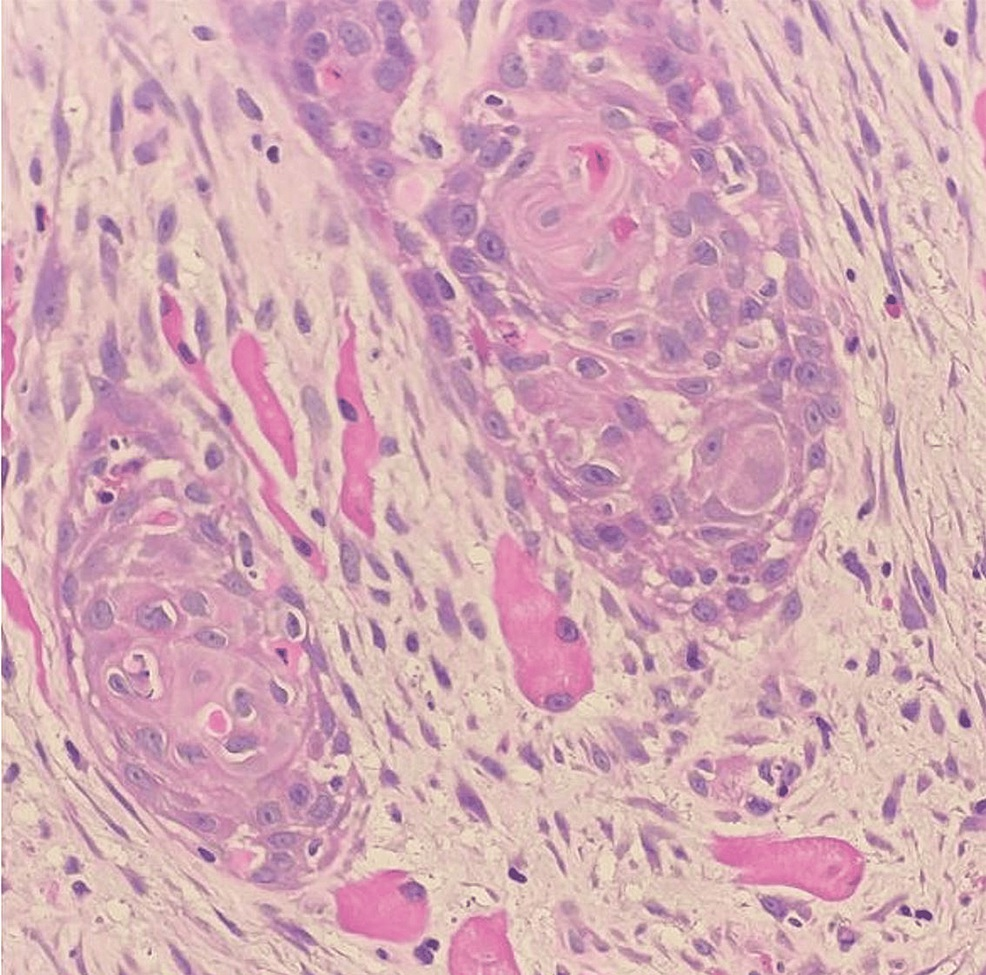
Moderately differentiated squamous cell carcinoma Histological findings of polygonal tumor cells displaying mildly pleomorphic nuclei, prominent nucleoli, and abundant eosinophilic cytoplasm. Intercellular bridges, individual cell keratinization, and dyskeratotic cells are present. (H&E with 400X magnification).

**Figure 8:**
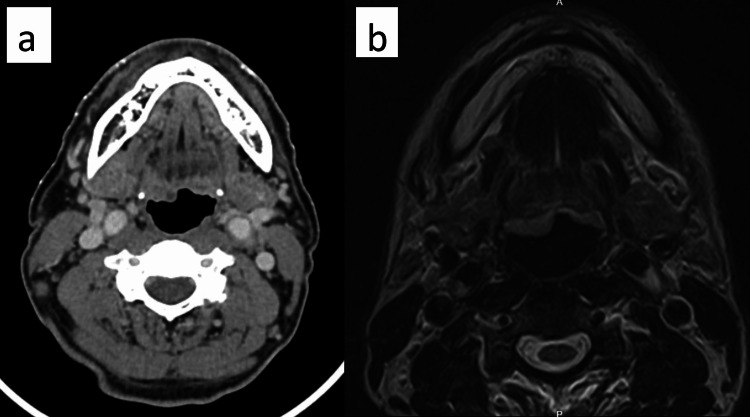
a: The CT scan showed thickening at the right palatine tonsil, retromolar trigone, and anterior and posterior tonsillar pillars. b: MRI showed an ill-defined lesion at the right retromolar trigone, laterally involving the right buccinator muscle.

The histopathological analysis revealed the presence of moderately differentiated squamous cell carcinoma (Figures 6, 7), characterized by a negative expression of p16. The MRI report showed heterogeneous enhancement of the right palatine tonsil, retromolar trigone, and anterior and posterior tonsillar pillar with loss of plane with the base of the tongue and a poor plane seen with the right medial pterygoid muscle (Figure 8). CT staging showed no evidence of distant metastasis. He was then diagnosed with oropharyngeal cancer with a staging of T4N0M0. He received counseling and expressed a strong interest in undergoing surgical intervention. He had planned for direct laryngoscopy, contact endoscopy, wide local excision, bilateral tonsillectomy, and neck dissection. Regrettably, he defaulted on his follow-ups and surgery.

**Figure 9:**
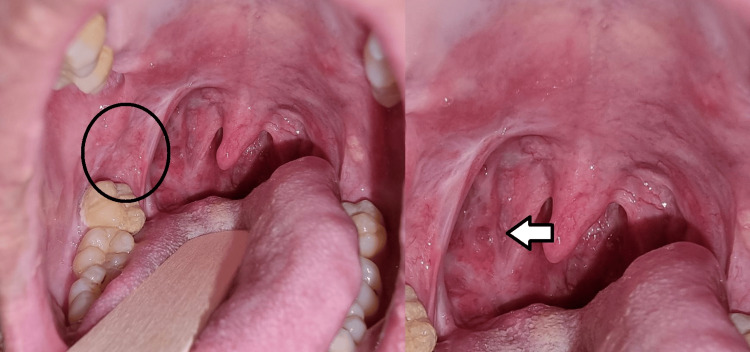
Fullness and erythematous at the right retromolar trigone area as shown in the black circle in the figure The right tonsillar fossa also appears erythematous as shown by the arrow. Fibrotic changes are seen over the previous biopsy sites as well.

**Figure 10:**
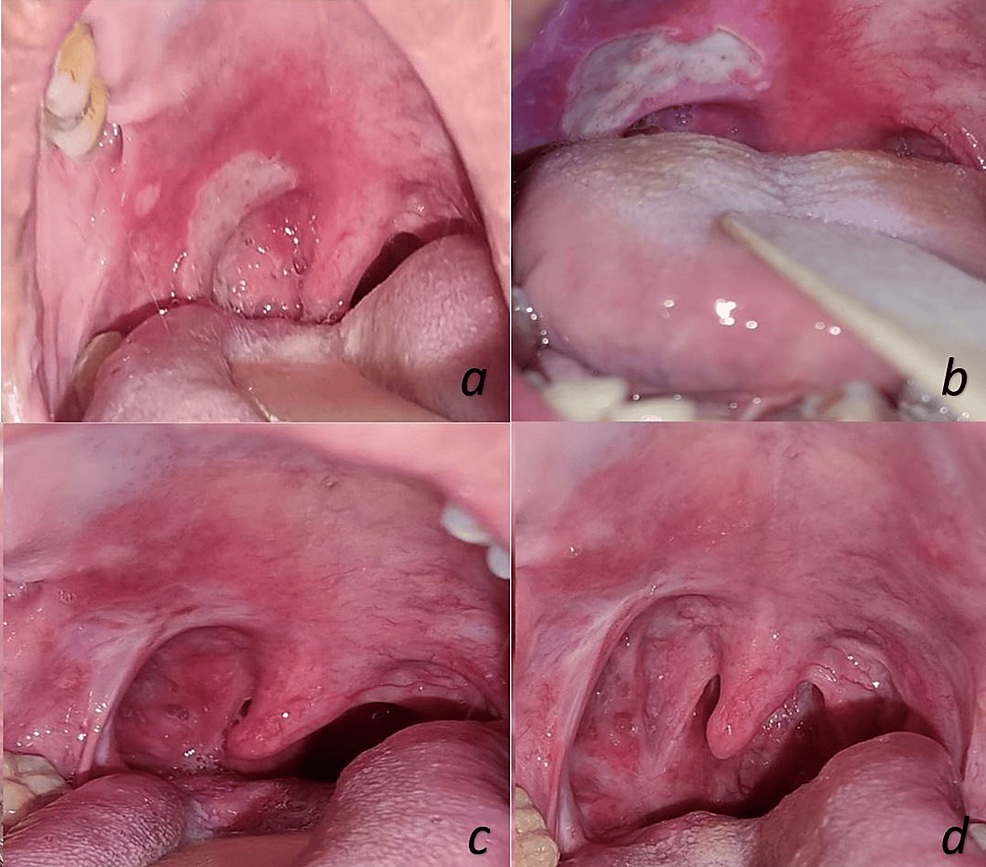
Intraoral examination at: a: initial presentation, b: one-month post-medical treatment, c: one-month post-biopsy, d: five-month post-biopsy

He presented back to us four months after his most recent follow-up appointment, with deteriorating symptoms that were identical. The present intraoral examination revealed a novel fullness in the right retromolar trigone region, previously not observed in previous follow-ups (Figures 9, 10). Other physical examinations were unremarkable.

He underwent repeated staging imaging, which showed findings similar to previous staging scans with no metastasis. Subsequently, he was counseled for surgical intervention in view of his immunohistochemistry biopsy result showing negative p16 expression. However, he expresses a strong desire for an oncology referral to pursue additional medical interventions.

## Discussion

The World Health Organization (WHO), in its Globocan Report 2020, estimated that the global burden of cancer was 19.3 million new cases, 9.9 million cancer deaths, and 50 million people living with cancer within five years of diagnosis [1]. Accordingly, the author of this case report aims to emphasize the significance of identifying the warning signs of patient symptoms and signs that may indicate a malignant origin. Typically, the onset of oral cancer manifests as a discreet erythematous or leukoplakic lesion or ulceration within the mouth cavity. It can impact many regions within the oral cavity, such as the tongue, lips, buccal mucosa, floor of the mouth, gingiva, or palate [2]. Generally, oral mucosal turnover should occur in <10 days; therefore, any persistent ulcer present for ≥2 weeks should be referred to a specialist for a biopsy.

Squamous cell carcinoma is the predominant form of neoplasia that primarily affects the mucosal lining of the upper aerodigestive tract, constituting approximately 89% of all diagnosed cancer types within this region [3]. In Malaysia, it has been observed that oral squamous cell carcinoma accounts for approximately 10.6% of deaths in government hospitals [4]. Furthermore, a significant proportion of these cases (67.1%) are identified at an advanced stage [5] due to the lack of awareness and understanding [6].

Herein, this case highlights the significance of regular follow-up, particularly for one with a strong suspicion of a malignant diagnosis, even if the blood parameters and histopathological examination results indicate otherwise. This patient's final conclusive HPE under general anesthesia reveals a case of oropharyngeal squamous cell carcinoma with a negative p16 status. Based on his p16 status, he received initial counseling for a surgical intervention. Regrettably, he failed to fulfill his commitment to follow-up, and subsequent attempts to reach him were unsuccessful. He returned to us, but this time, he agreed to an oncology referral. Based on his negative p16 status, it is advisable for surgical intervention, but the patient was not keen.

Patient awareness and education is paramount. This can be emphasized via regular screening programs. Oral cancer screening is one of the cancer screening programs offered by Malaysia's National Strategic Plan for Cancer Control Program. The screening protocol for oral cancer entails conducting oral examinations on individuals aged 18 years and older who are known to engage in high-risk activities, such as oral sex and open-mouthed kissing, or reside in a neighborhood with a higher likelihood of adopting such habits. The high-risk communities highlighted comprise the Indian community residing in rubber and palm oil estates in Peninsular Malaysia and other Bumiputera communities in Sabah and Sarawak [7].

Within Malaysia's population, another study documented a disparity of this disease across different provinces within Peninsular Malaysia. The state of Selangor exhibited the most significant incidence rate of oral cancer, with a prevalence of 8.2 cases per 100,000 individuals. Conversely, Kelantan, Kedah, and Terengganu states demonstrated the lowest incidence rates, with a prevalence of 1.9 cases per 100,000 individuals [8]. Among the major ethics groups in Malaysia, there are differences among different ethnic groups, particularly those of multiracial backgrounds. It was shown that approximately half of the reported instances in Malaysia were observed among individuals of Indian descent [9]. Another study also highlights the prevalence was highest among individuals of Indian ethnicity (63.8%), followed by Malays (19.6%) and Chinese (16.6%) [10].

The high prevalence of betel quid chewing among Indians may contribute to the increased occurrence, indicating that lifestyle and cultural practices can predispose individuals to this condition [11]. The cultural practices of alcohol intake, betel quid chewing, and tobacco smoking have been identified as established risk factors within the field of oral health. These practices are connected with developing pre-malignant oral lesions and oral squamous cell carcinoma [12].

While risk factors, such as tobacco and alcohol intake, continue to play a significant role in the development of oropharyngeal squamous cell carcinoma (OPSCC), it has become evident that oncogenic human papillomavirus (HPV) is also a crucial etiological component [13]. The prevalence of OPSCCs linked to human papillomavirus (HPV) in Malaysia seems to be comparatively lower when compared to European and American populations. However, it is plausible that the incidence of HPV-associated OPSCCs would be higher among individuals of Chinese ancestry in Malaysia [14].

Effective collaboration between clinicians and pathologists is crucial. Given its significant function, p16 is a crucial factor in determining the result of managerial decisions. Incorporating P16 immunohistochemistry is crucial for a comprehensive histological evaluation. HPV-positive OPSCC is currently acknowledged as a clinically and pathologically different subtype of head and neck squamous cell carcinoma (HNSCC). It exhibits unique demographic, clinical, and morphological characteristics associated with better clinical outcomes [5].

Individuals with tumors that test positive for human papillomavirus (HPV) are commonly characterized by a lack of smoking history and a slight intake of alcohol. There seems to be a correlation between sexual behavior, namely, the early age of sexual debut and a growing number of sex partners, and the occurrence of HPV-related OPSCC [15]. However, for the patient in this case report, the p16 was negative. This corresponded to his social risk factors, which are absent as described above. Thus, according to his initial staging, surgical intervention was counseled.

The paper also discusses the significance of follow-up in the early detection, treatment, and comprehensive support required to meet the distinct difficulties encountered by individuals with oropharyngeal cancer. Providing effective follow-up care for oropharyngeal cancer requires a patient-centered approach. Open communication, collaborative decision-making, and customizing follow-up plans based on specific patient requirements to improve overall satisfaction and adherence to the care regimen [16].

This case illustrates the challenge of managing a patient who defaults on his follow-up. Defaulting is a significant concern within the various fields of medicine, with particular relevance to cancer. Defaulting care as a cancer patient should be managed thoughtfully and require a multilevel approach. It is crucial for physicians to establish a secure environment for patients to receive counseling. By identifying the condition early and prioritizing the patient's needs, follow-up care will improve outcomes and boost the patient's compliance. Another study indicates that providing psychological support to those who default on cancer treatment may lead to higher acceptance rates of therapy and potentially enhance overall survival outcomes [17].

## Conclusions

This case report intends to contribute to the current knowledge by highlighting the importance of recognizing the clinical presentation of oropharyngeal cancer for the readers. According to current knowledge, a non-healing ulcer should be investigated as a potential cancer, as a diagnosis will be achieved by a simple biopsy without the diagnostic dilemma. Nevertheless, this case contributes to the existing knowledge by presenting a case report where cancer remains undiagnosed until the very end, despite the use of appropriate biopsy and investigation methods. This case also contributes to the existing information by emphasizing that medical practitioners exercise greater caution and vigilance when managing such a case and consider referring the patient for further evaluation if there is a genuine suspicion of cancer, especially in primary care. The manifestation of a non-healing ulcer may prompt clinicians to exercise caution when considering the diagnosis. If appropriate therapeutic measures have been implemented and symptoms persist, the clinicians must conduct a thorough investigation with the greatest priority to establish an accurate diagnosis. In this particular scenario, it is imperative to prioritize the diagnosis of malignancy and strongly recommend a referral for biopsy to establish the diagnosis definitively. This instance also underscores the importance of considering cancer as a potential cause when an initial presentation of illness fails to respond to medical treatment. The accuracy of the treating clinicians' judgment and decision-making is crucial, as the appropriate care of an oral ulcer caused by infection differs significantly from the management of oropharyngeal cancer despite the potential commonality in presenting symptoms. Ensuring appropriate counseling and subsequent monitoring is paramount to avoid delays in diagnosis and provide timely treatment in such circumstances, as timely management is crucial in malignancy cases.
